# Optimal Low-Frequency Parameter of Percutaneous Electrical Nerve Stimulation in Patients with Lower Back Pain: A Pilot Study

**DOI:** 10.3390/life15071005

**Published:** 2025-06-25

**Authors:** Roberto San-Emeterio-Iglesias, Carlos Romero-Morales, Francisco Minaya-Muñoz, Blanca De-la-Cruz-Torres

**Affiliations:** 1 Department of Physiotherapy, University Gimbernat-Cantabria, Aurelio García Cantalapiedra Street, s/n, 39300 Torrelavega, Spain; roberto.sanemeterio@eug.es; 2 Department of Physiotherapy, Faculty of Medicine, Health and Sports, European University of Madrid, Villaviciosa de Odón, 28670 Madrid, Spain; 3 MVClinic Institute, 28600 Madrid, Spain; franminaya@mvclinic.es; 4 Department of Physiotherapy, CEU San Pablo University, 28925 Madrid, Spain; 5 Department of Physiotherapy, University of Seville, Avicena Street, 41009 Seville, Spain

**Keywords:** nerve stimulation, low-frequency parameters, pain, low back

## Abstract

**Background**: The methodology of ultrasound (US)-guided percutaneous neuromodulation (PNM) remains unclear. **Objective**: To determine the optimal stimulation frequency (3 Hz vs. 10 Hz) during the short-term application of US-guided PNM on the sciatic nerve, we assessed the therapeutic benefits, including pain reduction, hip passive internal rotation range of motion (IR-ROM), balance, and functionality, in patients with chronic low back pain (LBP). **Methods**: Forty patients with LBP were randomly assigned to two groups, each receiving isolated percutaneous electrical stimulation of the sciatic nerve. One group received stimulation at 3 Hz, while the other received stimulation at 10 Hz. Pain intensity, hip passive IR-ROM, hip muscle strength, and the Oswestry Disability Index (ODI) were measured before and one week after the intervention. **Results**: Both interventions significantly reduced pain and improved ROM, balance, and functionality after one week (*p* = 0.001). However, significant between-group (treatment × time) differences were observed for pain intensity (*p* = 0.001) and flexion strength in the non-intervention limb (*p* = 0.01), though the effect size was small (η^2^ = 0.1). **Conclusions**: US-guided PNM applied to the sciatic nerve was more effective at 3 Hz than at 10 Hz in relieving pain intensity in patients with LBP.

## 1. Introduction

Chronic low back pain (LBP) is one of the most frequent reasons for medical consultation and is currently recognized as a major public health issue due to its high prevalence and socioeconomic impact [1,2]. It represents the leading cause of disability worldwide and significantly contributes to productivity loss [3].

The development and persistence of chronic LBP involve a complex interplay of biological, psychological, and social factors. Structurally, degenerative changes in the intervertebral discs, facet joints, or sacroiliac joints, as well as myofascial dysfunction and muscular imbalances, may contribute to nociceptive input [4,5]. Biomechanical alterations, including impaired lumbopelvic motor control and reduced trunk stability, have also been implicated in symptom perpetuation [6].

Beyond structural factors, psychological variables such as catastrophizing, fear-avoidant beliefs, depression, and anxiety are strongly associated with increased pain intensity and disability levels [4,7]. Furthermore, chronic LBP is increasingly recognized as a condition involving central nervous system dysregulation, with growing attention directed toward the contribution of neural mechanisms to its persistence [8]. As a result, neuromodulation techniques are gaining relevance for their potential to provide superior pain relief compared to conventional therapeutic approaches.

Peripheral nerve stimulation (PNS) has shown proven efficacy in the treatment of chronic neuropathic pain [7], including pain localized in the lumbar region [8,9]. The mechanism involves the use of low-frequency electrical currents to inhibit nociceptive afferent signals at the dorsal horn of the spinal cord [10,11], thereby producing an analgesic effect. In the field of physiotherapy, neuromodulation includes various non-invasive and minimally invasive techniques, such as transcutaneous electrical nerve stimulation (TENS), percutaneous electrical nerve stimulation (PENS), and electroacupuncture. All of these methods aim to modulate neural activity and reduce pain perception [12,13,14].

Currently, a novel therapeutic approach, known as ultrasound (US)-guided percutaneous neuromodulation (PNM), has emerged [14]. This minimally invasive technique is a variant of percutaneous nerve stimulation that involves delivering electrical stimulation via a needle electrode precisely positioned near a peripheral nerve or at the motor point of a muscle under ultrasound guidance. US-guided PNM has demonstrated promising analgesic effects in various chronic conditions, including chronic low back pain (LBP) [15,16], chronic lateral epicondylalgia [17], and anterior knee pain [18]. Beyond pain relief, this intervention has also shown improvements in range of motion (ROM), muscle performance, and overall functionality [19,20,21,22]. Consequently, several authors advocate viewing US-guided PNM not solely as an analgesic modality but as a comprehensive treatment that is capable of enhancing multiple functional outcomes. The increasing adoption of this technique is largely attributable to its clinical success; however, questions remain regarding the optimal application parameters to maximize therapeutic benefits. For example, San-Emeterio-Iglesias et al. [15] reported that the anatomical site of neuromodulation along the sciatic nerve pathway did not significantly affect clinical outcomes in patients with chronic LBP. Additionally, García-Bermejo et al. [18] observed a cross-over effect of US-guided PNM, suggesting systemic or contralateral neuromodulatory influences.

Despite these promising findings, the influence of stimulation frequency on therapeutic efficacy has not been sufficiently explored. Previous studies on electroacupuncture suggest that low-frequency stimulation (2–10 Hz) is more effective in modulating persistent inflammatory pain than high-frequency stimulation (e.g., 100 Hz) [23]. However, the range of values within the low-frequency spectrum is broad, and studies have used diverse frequencies such as 2 Hz [24,25], 3 Hz [15,16], and 10 Hz [17,18,20]. Evidence suggested that different frequencies within the low-frequency range may elicit distinct neurochemical responses. Specifically, low-frequency stimulation (e.g., 2 Hz) has been associated with endorphin-mediated analgesia. In contrast, higher frequencies within the low-frequency spectrum (e.g., 10 Hz) may preferentially promote the release of dopamine and enkephalins [26,27]. These frequency-specific neurophysiological effects support clinical observations indicating that variations in stimulation parameters can result in differential outcomes regarding pain modulation and treatment tolerance, potentially leading to distinct therapeutic responses.

Given the heterogeneity in study designs, stimulation parameters, and patient characteristics [28], the authors’ aim in this study was to clarify a methodological aspect related to the application of this technique, which may contribute to the establishment of standardized treatment protocols. Therefore, the main objective of this study was to compare the effects of two low-frequency protocols (3 Hz vs. 10 Hz) of US-guided PNM applied to the sciatic nerve in patients with chronic LBP, with pain intensity as the primary outcome. Secondary outcomes included hip ROM, balance, and functional capacity, aiming to determine the most effective stimulation frequency for short-term clinical improvement.

## 2. Materials and Methods

### 2.1. Design and Institutional Review Board

This study is a randomized controlled pilot trial (ClinicalTrials.gov Identifier: NCT05106920) conducted in accordance with the CONSORT guidelines. Prior to commencement, the protocol was approved by the local Institutional Review Board, adhering to the ethical principles outlined in the Declaration of Helsinki. All participants provided written informed consent before inclusion in the study.

### 2.2. Sample Size Calculation

Based on previous research by San-Emeterio-Iglesias et al. [15], where US-guided PNM was applied to a cohort of 30 individuals with chronic LBP and accounting for an estimated 20% dropout rate, a final sample size of 36 participants was deemed appropriate. To enhance statistical power, 40 participants were ultimately recruited.

### 2.3. Participants

**Figure 1:**
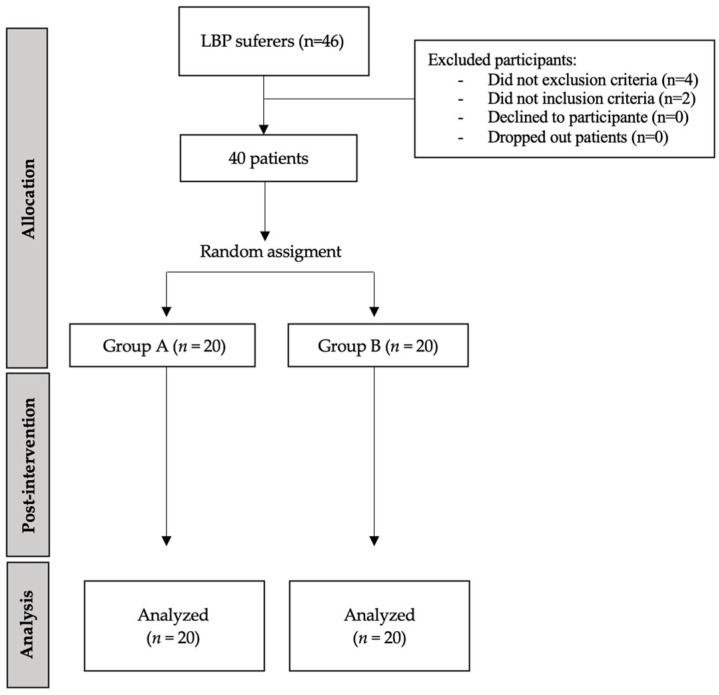
CONSORT flow diagram of subject recruitment for the study.

A total of 40 individuals diagnosed with chronic low back pain by an experienced physiotherapist at a private clinic were recruited between 3 November 2021 and 1 November 2023. The participants were randomly allocated into two groups (n = 20 per group) by an independent investigator using a computer-generated randomization program. Both participants and outcome assessors were blinded to group allocation, meaning they were unaware of the frequency range applied to each group. The participant flow throughout the study is detailed in Figure 1.

The inclusion criteria were as follows: aged between 18 and 65 years [29]; the presence of nonspecific low back pain (LBP) for at least the previous six months [30]; pain intensity ≥ 3/10 on the Numerical Rating Scale (NRS) [31]; and unilateral limitation of passive hip internal rotation. Chronic LBP has been associated with this specific movement impairment [32]. Specifically, studies have reported that individuals with chronic LBP often present with reduced hip internal rotation and side-to-side asymmetries, which may contribute to compensatory spinal loading and the persistence of symptoms [33,34,35].

The exclusion criteria were undergoing medical treatment or presenting with any musculoskeletal or neuropathic disorder, as confirmed by a qualified clinician. This was intended to ensure that the sample consisted exclusively of patients with nonspecific LBP, minimizing the confounding factors and allowing the intervention to be tested in the absence of identifiable structural or neurological conditions. Additional exclusion criteria included previous lower limb or lumbar spine surgery, electrophysiological alterations attributable to other peripheral nerve conditions, contraindications to electrical stimulation or needling [14], pregnancy, and epilepsy.

### 2.4. Outcome Measures

The age, weight, height, body mass index (BMI), sex, dominant side, and intervention side were registered as sociodemographic variables.

The clinical outcomes used in this study were as follows:

- Pain intensity in the lumbar region was measured using the numerical rating scale (NRS).
- Disability was assessed using the Oswestry Disability Index (ODI) [36], a validated 10-item questionnaire evaluating the impact of LBP on daily life. ODI scores are categorized as minimal (0–20), moderate (21–40), severe (41–60), crippled (61–80), or bed-bound/exaggerated symptoms (81–100).

The functional variables were as follows:

- Dynamic balance was assessed using the Y Balance Test (YBT), evaluating the anterior (ANT), posteromedial (PM), and posterolateral (PL) reach distances [37,38]. The values were recorded bilaterally in centimeters. Patients with chronic LBP typically present with reductions in the PM and PL reach directions [38].
- Passive hip internal rotation range of motion (IR-ROM) was assessed bilaterally using a universal goniometer [39]. The participants were placed prone with their knees flexed to 90°, hips in a neutral position, and pelvis stabilized with a belt. Two experienced raters performed the measurements following standardized procedures. Only internal rotation was assessed due to its documented relevance to LBP [40].
- Hip isometric muscle strength was evaluated for abduction, internal rotation, external rotation, flexion, and extension using a hand-held dynamometer (HHD) (Power Track II Commander; JTECH Medical, USA) [41]. Positions were selected based on clinical standards [42]: prone for extension, sitting for rotation, and supine for flexion and abduction. The participants were familiarized with submaximal isometric contractions prior to testing.

All assessments were conducted at baseline and one week after intervention by two experienced physiotherapists blinded to group allocation. Each functional test was performed three times per limb, and the average was used for analysis. The selected follow-up duration aligns with prior studies showing that the effects of US-guided PNM persist for up to one week [15,18].

### 2.5. Ultrasound-Guided PNM Intervention

Both groups received a single-session US-guided percutaneous neuromodulation intervention administered by an expert in invasive physiotherapy. The sciatic nerve was chosen as the target due to its anatomical origin in the lumbosacral plexus and its role in innervating the lower back and lower extremities.

**Figure 2:**
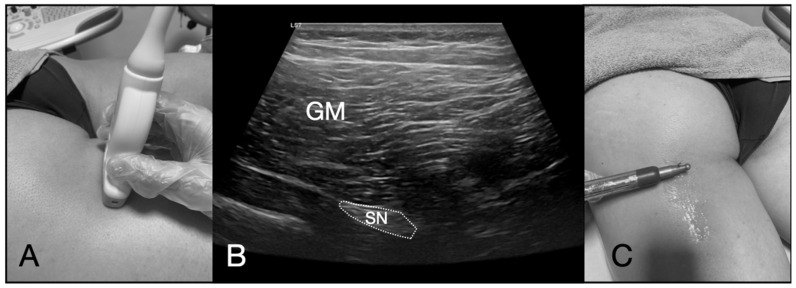
Ultrasound-guided percutaneous neuromodulation. (**A**) Proximal location on the sciatic nerve; (**B**) ultrasound image of the sciatic nerve; (**C**) electrical stimulation to the sciatic nerve using a needle. Abbreviations: GM, gluteus maximus; SN, the sciatic nerve.

After disinfecting the skin with chlorhexidine, an acupuncture needle (0.30 mm × 75 mm) was inserted using a long-axis in-plane approach, targeting the perineural space in the proximal posterior thigh (Figure 2). A high-frequency linear transducer (12 L) connected to a GE Logiq E^®^ ultrasound device (GE Healthcare, Wisconsin, USA) was used to guide the needle.

The electrical stimulation protocol followed prior studies [15,16], consisting of biphasic square wave currents; pulse width: 250 µs, with an intensity sufficient to elicit visible muscle contraction, and a stimulation dose of 10 repetitions of 10 s, each followed by 10 s of rest. Regarding the frequency parameter, the value of the frequency depended on the study group: the participants in Group A received an intervention with a 3 Hz frequency parameter, and the participants in Group B received an intervention with a 10 Hz frequency parameter. Stimulation was delivered using the Physio Invasiva^®^ device (CEO120; PRIM Physio, Madrid, Spain) in the PES mode. The participants were positioned prone, with their feet extending off the edge of the treatment table.

As in previous studies [15,16], the intervention was applied to the limb with reduced IR-ROM.

### 2.6. Data Analysis

All statistical analyses were performed using IBM SPSS Statistics for macOS, version 24.0 (Armonk, NY, USA). The normality of the data was verified using the Shapiro–Wilk test, assuming a normal distribution when the *p* > 0.05.

To examine the effects of the intervention, a two-way repeated measures ANOVA was conducted for each dependent variable, assessing both within-subject effects (time) and between-subject effects (group). Bonferroni correction was applied for the post hoc comparisons to control for type I errors. The effect size was calculated using eta squared (η^2^), interpreted according to the conventional thresholds (small ≥ 0.01, medium ≥ 0.06, large ≥ 0.14).

Statistical significance was set at *p* < 0.05. The analyses were conducted with a confidence level of 95% (α = 0.05) and a statistical power of 80% (β = 0.20).

## 3. Results

No statistically significant baseline differences were observed between the two groups in any demographic or anthropometric variables (Table 1), including age (42.4 ± 12.1 vs. 41.3 ± 10.9 years), weight (73.3 ± 13.2 vs. 68.9 ± 12.6 kg), height (1.72 ± 0.09 vs. 1.69 ± 0.08 m), and body mass index (24.6 ± 3.24 vs. 23.9 ± 3.08) (*p* > 0.05 for all comparisons). Distribution by sex (female/male: 6/14 vs. 12/8), dominant limb (right/left: 17/3 vs. 14/6), and intervention side (right/left: 9/11 vs. 11/9) was also documented. No adverse events were reported throughout the intervention period, as documented by the absence of any vasovagal reactions.

As shown in Table 2, the intra-group analysis (effect of time) revealed statistically significant improvements (*p* = 0.001) for all clinical outcome measures, including pain and disability [43]. Although the ODI showed significant intra-group improvement (*p* = 0.001; η^2^ = 0.76), the minimum clinically important difference (MCID) of 17 points was not reached. In both groups, the post-intervention ODI scores remained within the category of “minimal disability.”

In terms of inter-group differences (treatment × time interaction), significant differences were observed only for pain intensity (*p* = 0.001), favoring one of the intervention frequencies. No significant interaction effects were found for the other clinical outcomes.

Table 3 displays the results of the Y Balance Test (YBT). The intra-group analyses indicated statistically significant improvements in all directions (anterior, posteromedial, and posterolateral) for both limbs (*p* = 0.001). However, there were no significant inter-group (treatment × time) interactions, suggesting similar balance improvements regardless of the stimulation frequency.

As presented in Table 4, all hip strength variables showed significant intra-group improvements over time (*p* = 0.001). Nonetheless, inter-group differences were only found for hip flexion strength on the intervention side (*p* = 0.01), with a small effect size (η^2^ = 0.10).

## 4. Discussion

The main finding of this pilot study suggested that the choice of low-frequency parameter used in the application of ultrasound (US)-guided percutaneous neuromodulation (PNM) may influence the therapeutic outcomes in patients with chronic low back pain (LBP). While both 3 Hz and 10 Hz frequencies demonstrated beneficial effects on the clinical and functional variables, stimulation at 3 Hz was significantly more effective in reducing pain intensity, achieving a 60.82% reduction, compared to only 21.30% at 10 Hz (Table 2).

To the authors’ knowledge, this is the first study to directly compare different frequencies of US-guided PNM applied to the sciatic nerve in chronic LBP patients. Previous studies have demonstrated the effectiveness of PNM in various musculoskeletal pain conditions, such as chronic lateral epicondylalgia (10 Hz) [17], anterior knee pain (10 Hz) [18], and cubital tunnel syndrome (2 Hz) [25]. In LBP, stimulation at 3 Hz has shown promising results [15,16], but no comparative analyses have been conducted until now.

This study contributes to the growing body of evidence supporting US-guided PNM as a clinically valuable tool for chronic pain management, with pain relief potentially depending on the stimulation frequency used. These findings align partially with the results from other neuromodulatory therapies. For instance, Zhang et al. [23] reported that electroacupuncture at 2–10 Hz provides more effective pain relief than stimulation at 100 Hz. However, the wide spectrum of “low-frequency” values (2–10 Hz) remains underexplored in terms of specific outcomes. Similarly, Hernández et al. [24] found no differential effects between high and low-frequency PENS when combined with deep dry needling for myofascial neck pain, raising questions about the influence of stimulation alone versus technique synergy.

Additionally, prior research has highlighted several characteristics of US-guided PNM: its ability to exert cross-over effects [19], the versatility in stimulation points along a nerve without compromising therapeutic impact [15], and lasting effects for up to one week after a single intervention [18]. The present findings expand upon this knowledge by proposing frequency-specific guidelines for clinical practice.

### 4.1. Clinical Applications

From a clinical standpoint, this study reinforces the therapeutic potential of ultrasound-guided percutaneous neuromodulation (PNM), highlighting the importance of careful parameter selection, particularly stimulation frequency, to optimize patient outcomes. Clinicians are encouraged to base their application of the technique on current evidence and sound clinical reasoning, recognizing that stimulation frequency is not a trivial variable but one that may significantly influence analgesic efficacy.

### 4.2. Limitations and Future Directions

Despite the promising results, several limitations must be acknowledged as follows: 1. Unimodal approach: This study evaluated ultrasound-guided percutaneous neuromodulation (PNM) in isolation. Future research should explore its integration into multimodal treatment protocols, such as combinations with therapeutic exercise or manual therapy; 2. Protocol specificity: Only one PNM protocol was tested, despite previous studies employing various parameters (e.g., number of stimulations, pulse width, intensity). The influence of frequency should be evaluated across a broader range of PNM configurations; 3. Lack of a placebo group: Although the study included two active intervention groups, the absence of a control or sham group limits conclusions regarding potential placebo effects. However, given the short-term scope and pilot nature of this study, the two-group design was considered appropriate.

Despite these limitations, the findings offer preliminary evidence to guide future research, inform the development of standardized methodological recommendations, and support the clinical use of frequency-specific ultrasound-guided PNM protocols.

## 5. Conclusions

The findings of this pilot study indicated that stimulation at 3 Hz is more effective than at 10 Hz in achieving short-term pain relief. These preliminary results suggest that frequency selection is a key parameter in optimizing the clinical outcomes of US-guided PNM and should be considered carefully in both research and clinical practice.

## Figures and Tables

**Table 1:** Demographic and anthropometric characteristics of the two groups.

Variable	Group A (3 Hz)	Group B (10 Hz)	*p*-Value
Age (years)	42.4 ± 12.1	41.3 ± 10.9	>0.05
Weight (kg)	73.3 ± 13.2	68.9 ± 12.6	>0.05
Height (m)	1.72 ± 0.09	1.69 ± 0.08	>0.05
Body mass index (kg/m^2^)	24.6 ± 3.24	23.9 ± 3.08	>0.05
Sex (female/male)	6/14	12/8	—
Dominant limb (right/left)	17/3	14/6	—
Intervention side (right/left)	9/11	11/9	—

Note: Values are expressed as mean ± standard deviation or as frequency counts. No statistically significant differences were observed between groups in any of the variables (*p* > 0.05 for all comparisons).

**Table 2:** Pain, Oswestry Disability Index (ODI), and internal rotation range of motion (IR-ROM) for both limbs intrasubject effects.

			Intrasubject Effects	
Measure	Group A (3 Hz)	Group B (10 Hz)	Time Value F; p (Eta^2^)	Treatment × Time F; p (Eta^2^)
**Pain (NRS)**			F = 94.6; P = 0.001 (0.713)	F = 18.2; P = 0.001 (0.324)
Baseline	4.85 ± 1.3	5.40 ± 1.4		
1 week	1.90 ± 1.2	4.25 ± 1.6		
**ODI (%)**			F = 120.49; P = 0.001 (0.760)	F = 3.21; P = 0.081 (0.078)
Baseline	16.3 ± 5.28	19.8 ± 5.69		
1 week	8.10 ± 4.88	13.9 ± 6.73		
**IR Sitting Non-Intervention limb (º)**			F = 18.8968; P = 0.001 (0.332)	F = 0.0992; P = 0.754 (0.003)
Baseline	37.4 ± 5.13	37.1 ± 5.72		
1 week	39.2 ± 3.78	38.8 ± 4.91		
**IR Sitting Intervention limb (º)**			F = 54.542; P = 0.001 (0.589)	F = 0.334; P = 0.567 (0.009)
Baseline	36.0 ± 5.13	35.8 ± 6.00		
1 week	39.1 ± 4.85	38.5 ± 5.08		

Values are mean ± SD unless otherwise indicated. Abbreviations: IR, internal rotation; NRS, numerical rating scale; ODI, Oswestry Disability Index; F, F-test; Df, degrees of freedom.

**Table 3:** Y Balance Test (YBT) for both limbs intrasubject effects.

			Intrasubject Effects	
Measure (Centimeters, cm)	Group A (3 Hz)	Group B (10 Hz)	Time Value F; p (Eta^2^)	Treatment × Time F; p (Eta^2^)
**Anterior Non-Intervention limb**			F = 160.732; P = 0.001 (0.809)	F = 0.373; P = 0.545 (0.010)
Baseline	54.6 ± 4.43	54.8 ± 4.76		
1 week	57.4 ± 4.87	56.3 ± 5.63		
**Anterior Intervention limb**			F = 28.94; P = 0.001 (0.432)	F = 2.78; P = 0.104 (0.068)
Baseline	54.4 ± 4.44	54.7 ± 6.54		
1 week	57.8 ± 4.23	56.4 ± 6.16		
**Posteromedial Non-Intervention limb**			F = 40.11; P = 0.001 (0.513)	F = 4.91; P = 0.053 (0.114)
Baseline	99.3 ± 11.9	96.2 ± 8.71		
1 week	108 ± 10.6	103 ± 9.40		
**Posteromedial Intervention limb**			F = 51.725; P = 0.001 (0.576)	F = 0.926; P = 0.342 (0.024)
Baseline	101 ± 12.5	97.0 ± 8.85		
1 week	110 ± 11.5	104 ± 8.88		
**Posterolateral Non-Intervention limb**			F = 63.852; P = 0.001 (0.627)	F = 0.570; P = 0.455 (0.015)
Baseline	95.7 ± 12.5	95.3 ± 9.53		
1 week	104 ± 12.5	99.8 ± 9.34		
**Posterolateral Intervention limb**			F = 36.49; P = 0.001 (0.490)	F = 3.22; P = 0.081 (0.078)
Baseline	95.1 ± 10.2	94.5 ± 7.91		
1 week	104 ± 11.5	101 ± 7.95		

Values are mean ± SD unless otherwise indicated. Abbreviations: ER, external rotation; IR, internal rotation; F, F-test; Df, degrees of freedom.

**Table 4:** Flexion, extension, and external and internal rotation in the prone position, and abduction strength for both limbs’ intrasubject effects.

			Intrasubject Effects	
Measure (Kgf)	Group A (3 Hz)	Group B (10 Hz)	Time Value F (Df); p (Eta^2^)	Treatment × Time F (Df); p (Eta^2^)
**Flexion Non-Intervention limb**			F = 67.88; P = 0.001 (0.641)	F = 1.08; P = 0.305 (0.028)
Baseline	15.3 ± 3.03	13.9 ± 2.89		
1 week	17.8 ± 3.73	17.1 ± 4.05		
**Flexion Intervention limb**			F = 171.16; P = 0.001 (0.818)	F = 7.02; P = 0.012 (0.156)
Baseline	14.4 ± 3.36	13.0 ± 2.57		
1 week	17.8 ± 5.10	18.3 ± 3.43		
**ER-prone Non-Intervention limb**			F = 80.611; P = 0.001 (0.680)	F = 0.199; P = 0.658 (0.005)
Baseline	12.3 ± 2.93	10.5 ± 1.99		
1 week	13.5 ± 3.60	12.3 ± 2.11		
**ER-prone Intervention limb**			F = 65.62; P = 0.001 (0.633)	F = 2.35; P = 0.134 (0.058)
Baseline	11.6 ± 2.22	10.6 ± 2.17		
1 week	13.6 ± 3.37	13.6 ± 2.51		
**IR-prone Non-Intervention limb**			F = 82.74; P = 0.001 (0.685)	F = 2.50; P = 0.122 (0.062)
Baseline	10.6 ± 2.49	9.97 ± 1.52		
1 week	12.8 ± 3.28	12.1 ± 2.49		
**IR-prone Intervention limb**			F = 54.1368; P = 0.001 (0.588)	F = 0.0141; P = 0.906 (0.000)
Baseline	9.14 ± 2.36	9.10 ± 1.59		
1 week	12.8 ± 3.74	13.2 ± 2.74		
**Abduction Non-Intervention limb**			F = 65.958; P = 0.001 (0.634)	F = 0.171; P = 0.682 (0.004)
Baseline	12.6 ± 2.18	11.4 ± 1.62		
1 week	14.8 ± 3.51	13.4 ± 2.07		
**Abduction Intervention limb**			F = 65.958; P = 0.001 (0.634)	F = 0.171; P = 0.682 (0.004)
Baseline	11.3 ± 1.92	10.5 ± 1.52		
1 week	15.3 ± 3.68	15.8 ± 2.45		
**Extension Non-Intervention limb**			F = 177.78; P = 0.001 (0.824)	F = 3.63; P = 0.065 (0.087)
Baseline	19.4 ± 4.40	16.6 ± 3.11		
1 week	24.9 ± 9.50	22.5 ± 4.90		
**Extension Intervention limb**			F = 53.3473; P = 0.001 (0.584)	F = 0.0797; P = 0.779 (0.002)
Baseline	17.5 ± 4.68	16.2 ± 2.95		
1 week	24.7 ± 9.57	24.1 ± 5.30		

Values are mean ± SD unless otherwise indicated. Abbreviations: ER, external rotation; IR, internal rotation; F, F-test; Df, degrees of freedom.

## Data Availability

The data presented in this study are available on request from the corresponding authors. The data are not publicly available due to ethical condition.
